# Risk Factors for Colonization With Multidrug-Resistant Bacteria in Urban and Rural Communities in Kenya: An Antimicrobial Resistance in Communities and Hospitals (ARCH) Study

**DOI:** 10.1093/cid/ciad223

**Published:** 2023-07-05

**Authors:** Mark A Caudell, Charchil Ayodo, Teresa Ita, Rachel M Smith, Ulzii-Orshikh Luvsansharav, Ashley R Styczynski, Brooke M Ramay, Samuel Kariuki, Guy H Palmer, Douglas R Call, Sylvia Omulo

**Affiliations:** Paul G. Allen School for Global Health, Washington State University, Pullman, Washington, USA; Washington State University Global Health–Kenya, Nairobi, Kenya; Washington State University Global Health–Kenya, Nairobi, Kenya; Division of Healthcare Quality Promotion, US Centers for Disease Control and Prevention, Atlanta, Georgia, USA; Division of Healthcare Quality Promotion, US Centers for Disease Control and Prevention, Atlanta, Georgia, USA; Division of Healthcare Quality Promotion, US Centers for Disease Control and Prevention, Atlanta, Georgia, USA; Paul G. Allen School for Global Health, Washington State University, Pullman, Washington, USA; Center for Health Studies, Universidad del Valle de Guatemala, Guatemala City, Guatemala; Kenya Medical Research Institute, Nairobi, Kenya; Paul G. Allen School for Global Health, Washington State University, Pullman, Washington, USA; Washington State University Global Health–Kenya, Nairobi, Kenya; University of Nairobi Institute of Tropical and Infectious Diseases, Nairobi, Kenya; Paul G. Allen School for Global Health, Washington State University, Pullman, Washington, USA; Paul G. Allen School for Global Health, Washington State University, Pullman, Washington, USA; Washington State University Global Health–Kenya, Nairobi, Kenya; University of Nairobi Institute of Tropical and Infectious Diseases, Nairobi, Kenya

**Keywords:** antimicrobial resistance, public health, communities, Kenya

## Abstract

**Background:**

Colonization with antimicrobial-resistant bacteria increases the risk of drug-resistant infections. We identified risk factors potentially associated with human colonization with extended-spectrum cephalosporin-resistant Enterobacterales (ESCrE) in low-income urban and rural communities in Kenya.

**Methods:**

Fecal specimens, demographic and socioeconomic data were collected cross-sectionally from clustered random samples of respondents in urban (Kibera, Nairobi County) and rural (Asembo, Siaya County) communities between January 2019 and March 2020. Presumptive ESCrE isolates were confirmed and tested for antibiotic susceptibility using the VITEK2 instrument. We used a path analytic model to identify potential risk factors for colonization with ESCrE. Only 1 participant was included per household to minimize household cluster effects.

**Results:**

Stool samples from 1148 adults (aged ≥18 years) and 268 children (aged <5 years) were analyzed. The likelihood of colonization increased by 12% with increasing visits to hospitals and clinics. Furthermore, individuals who kept poultry were 57% more likely to be colonized with ESCrE than those who did not. Respondents’ sex, age, use of improved toilet facilities, and residence in a rural or urban community were associated with healthcare contact patterns and/or poultry keeping and may indirectly affect ESCrE colonization. Prior antibiotic use was not significantly associated with ESCrE colonization in our analysis.

**Conclusions:**

The risk factors associated with ESCrE colonization in communities include healthcare- and community-related factors, indicating that efforts to control antimicrobial resistance in community settings must include community- and hospital-level interventions.

## Teaser

Colonization with extended-spectrum cephalosporin-resistant Enterobacterales in Kenya is associated with contact with healthcare facilities and poultry tending but not antibiotic use. Mitigation strategies for antimicrobial resistance in communities may be most effective if focused on the factors that promote transmission.

The prevalence of extended-spectrum cephalosporin-resistant Enterobacterales (ESCrE) is increasing in many communities despite their common association with healthcare settings [1–4]. ESCrE are resistant to cephalosporins, antibiotics that play an important role in controlling infections globally. In East Africa, ceftriaxone—a third-generation cephalosporin—is among the most commonly prescribed antibiotics in healthcare settings [5–10]. As resistance spreads, ESCrE transmission in healthcare facilities and communities will likely increase, leading to infections with fewer antibiotic treatment options. This will increase morbidity and mortality rates [11–13], medical costs, and demand for broader-spectrum and more expensive antibiotics [14, 15].

Most antimicrobial resistance data are drawn from infections detected in healthcare facilities, and surveillance programs rarely evaluate antibiotic resistance in communities [10]. Colonization with ESCrE in hospitals has been well described and is associated with ESCrE infections within hospital settings [4, 16–19], but there is less information about factors associated with ESCRE colonization in community settings [10]. Without these data, identifying the origins of and transmission pathways for these organisms, and potential interventions to limit colonization are difficult in these settings.

A previously published Antimicrobial Resistance in Communities and Hospitals (ARCH) study in Botswana reported a 24%–26% ESCrE colonization prevalence in communities [4], while a similar study in Kenya reported approximately 2-fold higher rates of colonization (45% rural, 52% urban) [16]. As a follow-up to the latter study, we used a path analytic model to explore the factors potentially associated with ESCrE colonization in a rural and urban community in Kenya.

## METHODS

### Study Design and Sample Analysis

This cross-sectional study, which happened between January 2019 and March 2020, was part of the ARCH studies conducted across 6 countries to evaluate the population-based prevalence of colonization with clinically significant antimicrobial-resistant organisms [10]. No deviations were made from the main ARCH protocol, and 3 target microorganisms were investigated. Prevalence data on the target microorganisms, including descriptions of the population demographics, antibiotic use measures, and microbiological procedures used, have been published elsewhere [16].

### Study Participants

Enrolled participants were part of an ongoing population-based infectious disease surveillance program [17] and came from 2 communities in Kenya, 1 rural (Asembo) and 1 urban (Kibera). Kibera is a high-density urban informal settlement in Nairobi County that has mostly poor-quality housing and inadequate garbage disposal and lacks formal sewers. Two villages in Kibera, Gatwekera and Soweto, served as the sampling frame. The rural community of Asembo, located in Siaya County, comprises 33 dispersed settlements where the primary sources of livelihood are subsistence farming and fishing [16].

Participating households were selected using a 2-stage cluster sampling design (random household selection and of individuals within households) [16]. Where available, a child (aged <5 years) and an adult (aged ≥18-years) were enrolled from each household. After written consent was obtained, a structured questionnaire was used to collect data on demographics, healthcare use, occupational exposures, animal contact, access to safe water and sanitation facilities, and recent food consumption.

### Data Collection and Processing

Participants provided freshly voided stool samples that were tested using commercial HardyCHROM extended-spectrum β-lactamase agar plates [16]. Up to 3 morphologically distinct colonies were identified from each plate and characterized using a bioMérieux Vitek2 instrument (GN ID and AST-GN71 cards). Bacterial isolates identified as Enterobacterales were classified as ESCrE if resistant to ceftriaxone and as susceptible or intermediate to all the carbapenems tested (ertapenem, imipenem, and meropenem).

For analysis, a person was considered ESCrE colonized if ≥1 isolate collected from their stool sample met the criteria for ESCrE classification. A logistic regression path model [20] was used to partition the variance between the factors that directly or both directly and indirectly affect the risk of colonization with ESCrE. For example, healthcare contact could directly affect colonization if visits to a hospital or clinic result in a higher probability of ESCrE transmission through interaction with the healthcare environment [18, 19]. It can indirectly affect ESCrE colonization by increasing antibiotic use and downstream selection effects on resistance (see graphic representation in Supplementary Figure 2).

**Figure 1.**
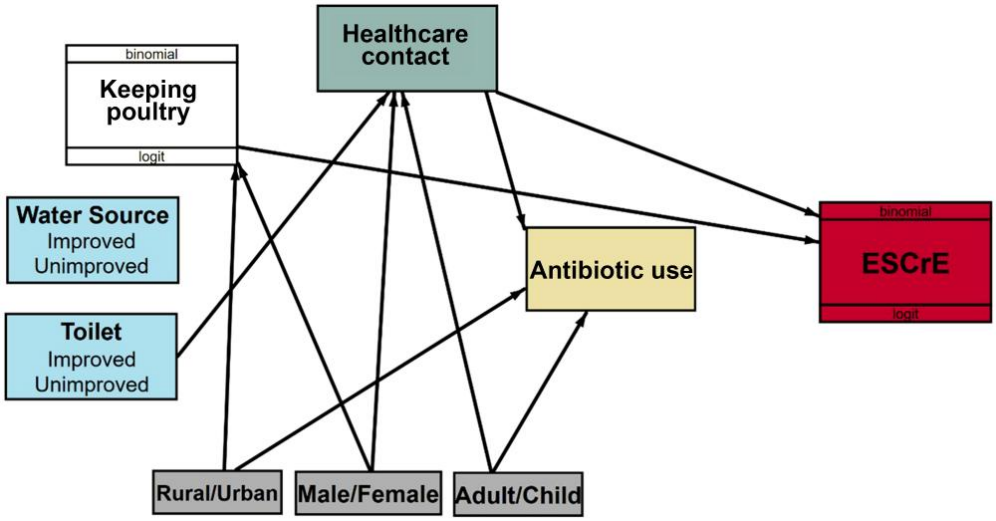
Path model diagram. Statistically significant (*P* < .05) relationships are shown as arrows and indicate that an outcome variable was regressed on an independent variable. Supplementary Table 1 provides a rationale for the hypothesized relationships that were tested with this model, and Supplementary Table 2 shows other nonsignificant relationships that were considered in the model. Abbreviation: ESCrE, extended-spectrum cephalosporin-resistant Enterobacterales.

To construct the path model, variables that were potentially related to bacterial transmission and selection of antibiotic-resistant bacteria based on literature review (eg, antibiotic use) and field observations were included. Potential indirect pathways through which these variables might affect the probability of ESCrE colonization (eg, poor sanitation could lead to increased risk of illness that could increase demand for antibiotics; Table 1 and Figure 1) were also considered. Supplementary Table 1 and Supplementary Figure 1 provide a rationale for selecting potential direct and indirect risk factors for ESCrE colonization. With respect to poultry, a composite variable for household animal ownership was used initially, but after finding a significant relationship the variable was decomposed leaving poultry keeping as the only significant animal-related variable.

**Table 1.** Description of Variables Used in the Path Model

Variable^a^	Definition
ESCrE	Participant’s stool sample negative (0) or positive (1) for ESCrE (results confirmed by Vitek2 assay)
Antibiotic use	Continuous variable indicating reported weeks of antibiotics use in the last 3 m
Healthcare contact	Continuous scaled variable combining answers to 4 healthcare questions about numbers of hospital or clinic visits for medical or nonmedical reasons within the last 6 m; responses to the questions were summed and scored as 0 (never), 1 (1–2 times), 2 (3–4 times), or 3 (≥5 times), with 12 the maximum possible value
Poultry keeping	Household kept poultry (1) or did not (0)
Toilet facilities	Household toilet facilities were unimproved (0) or improved (1)^b^
Water source	Household water source was unimproved (0) or improved (1)^b^
Urban/rural residence	Household location was urban (0) or rural (1)
Sex	Participant was male (0) or female (1)
Age	Participant was an adult (0) or a child (1)

Abbreviation: ESCrE, extended-spectrum cephalosporin-resistant Enterobacterales.

Supplementary Table 1 and Supplementary Figure 1 provide additional details about the selection of variables and their hypothesized relationships.

For household toilet facilities and water source, the categories were defined by the Joint Monitoring Program for Water Supply and Sanitation [21]. For toilet facilities, the “improved-limited” and “improved” classifications were combined, as most facilities meeting the improved criteria were shared among households.

Before the assessment of risk factors, potential design effects from the 2-stage cluster sampling design were accommodated [16]. Randomly selected households—the primary sampling units—were stratified by rural and urban settings. Randomly selected individual adults and children were treated as secondary sampling units, weighted according to the number of eligible individuals at each household. The *svyset* command in Stata software, version 17 [16], was used to specify the probability weights and strata, and the *svy* prefix was used before structural equation modeling commands (ie, *gsem*). Design effects (Supplementary Table 2) were estimated for each element in the final model (*estat effects* command).

For many households, sampling both an adult and child produced a statistically significant intraclass correlation coefficient (0.13 [95% confidence interval [CI], .04–.39]) indicating clustering at the household level; that is, individuals from the same household were 13% more similar in their likelihood of ESCrE colonization than those from different households. Consequently, where 2 household members were sampled, 1 individual was randomly removed (using the *runiform* command), so that each household was defined by the ESCrE status of a single individual. To determine whether patterns of random removals affected model results, the final model was run with 500 different random removals. Data cleaning, analysis, and post-estimation analysis were conducted using Stata software, version 17.

### Ethical Considerations

Human subject approvals were granted by the Kenyatta National Hospital/University of Nairobi (ethics review committee no. P164/03/2018), with reliance from Washington State University (institutional review board no. 16742-001), the Kenya Medical Research Institute and the Centers for Disease Control and Prevention (no. 7111). This project was licensed by the Kenya National Commission for Science, Technology and Innovation (no. NACOSTI-P-21-12461).

## RESULTS

A total of 1611 households and 1715 individuals were enrolled, of which 1416 individuals were retained for analysis, after removal of some individuals for incomplete data and random removal to ensure that only 1 person from each household was included (Table 2). The median age (interquartile range) was 2 (1–3) years in children and 35 (18–52) years in adults. The median household size was 5 members (interquartile range, 3–6).

**Table 2.** Descriptive Statistics for Variables Included in the Models

	ESCrE positive (n=688)			ESCrE negative (n=728)		
	Mean	SE	Min-max	Mean	SE	Min-max
**Antibiotic use (weeks)**	0.56	2.5	0-51.43	0.69	2.16	0-14
**Healthcare contact** ^a^	9.35	2.82	0-16	8.82	2.36	1-17
	Freq.	Percent	SE	Freq.	Percent	SE
**Keep poultry**			0.46			0.47
No	204	29.65		245	33.51	
Yes	484	70.35		483	66.49	
**Toilet risk**			0.45			0.48
Unimproved^b^	202	29.36		252	34.62	
Improved	486	70.64		476	65.38	
**Water risk**			0.5			0.5
Unimproved^b^	396	57.56		368	50.55	
Improved	303	42.44		360	49.45	
**Residence**			0.49			0.5
Urban	403	58.58		377	51.79	
Rural	285	41.42		351	48.21	
**Sex**			0.5			0.45
Male	206	29.94		200	27.47	
Female	482	70.06		528	72.53	
**Age^a^**			0.4			0.39
Adult	553	80.38		595	81.73	
Child	135	19.62		133	18.27	

Of the 8 variables included duration of antibiotic use and healthcare contact are continuous variables. The SEs are linearized SEs based on a first-order Taylor series linear approximation [20]. Data are shown for 1416 individuals.Abbreviations: ESCrE, extended-spectrum cephalosporin-resistant Enterobacterales; SE, standard error.

Values represent scores on healthcare contact scale, not absolute numbers of visits.

For household toilet facilities and water source, the categories were defined by the Joint Monitoring Program for Water Supply and Sanitation [21].

### Path Model

#### Contact With Healthcare Facilities and Keeping Poultry Are Associated With Colonization

We assessed associations between 8 variables and the probability of ESCrE colonization (Figure 1, arrows pointing to the ESCrE box), of which 2 variables were statistically significant (Table 3). For every unit increase in the healthcare contact scale (a continuous variable; Supplementary Table 3), the odds of colonization were 1.12 [1.03, 1.21] times greater. From the lowest level of healthcare contact (≤4 visits to a hospital and/or clinic in the past 6 months) to the highest (≥15 visits), the predicted probability of ESCrE colonization increased by approximately 40%–70% (Supplementary Figure 2). Furthermore, respondents whose households kept poultry (a binary variable) had higher odds of ESCrE colonization (odds ratio, 1.57 [95% CI, 1.18–2.07]).

**Table 3.** Path Model Results

Variable	Keeping Poultry, OR (95% CI)	Healthcare Contact, β Coefficient (95% CI)	Antibiotic Use, β Coefficient (95% CI)	ESCrE Colonization, OR (95% CI)
Antibiotic use	…	…	…	0.95 (.89–1.02)
Healthcare contact	…	…	0.11 (.05–.18)^a^	1.12 (1.03–1.21)^a^
Keeping poultry (yes = 1)	…	−0.19 (−.39 to .01)	−0.08 (−.30 to .13)	1.57 (1.18–2.07)^a^
Toilet facilities (improved = 1)	…	0.22 (.02–.42)^b^	−0.18 (−.46 to .11)	1.13 (.86–1.5)
Water risk (improved = 1)	…	−0.12 (−.35 to .11)	0.18 (−.05 to .4)	0.84 (.62–1.15)
Residence (rural = 1)	5.64 (4.20–7.5)^a^	0.12 (−.12 to .36)	0.56 (.33–.79)^a^	0.82 (.59–1.15)
Sex (female = 1)	1.4 (1.03–1.9)^b^	0.51 (.32–.7)^a^	−0.16 (−.42 to .11)	0.80 (.61–1.05)
Age group (child = 1)	0.75 (.51–1.09)	−0.42 (−.61 to .22)^b^	−0.34 (−.50 to .17)^a^	1.04 (.75–1.44)
Constant	0.97 (.73–1.31)	5.46 (5.18–5.74)^a^	−0.06 (−.46 to .34)	0.47 (.27–.84)^b^

This table shows the effects of 8 independent variables (first column) on ESCrE colonization (last column). The remaining columns show results for effects of different independent variables on other variables (keeping poultry, healthcare contact, and antibiotic use) when the latter are treated as dependent variables (Figure 1). Coefficients (and 95% CIs) for the continuous dependent variables of healthcare contact and antibiotic use are interpreted as β coefficients in linear regression. Coefficients associated with binary dependent variables of ESCrE positivity and keeping poultry are interpreted as ORs. See Table 1 for definitions of variables. Data from 1416 individuals were used in this model.

Abbreviations: CI, confidence interval; ESCrE, extended-spectrum cephalosporin-resistant Enterobacterales; OR, odds ratio.

*P* < .01.

*P* < .05.

### Colonization and Antibiotic use

In the path model, the odds of ESCrE colonization were not associated with a respondent's reported use of antibiotics (Table 3). Nevertheless, the duration of antibiotic use was associated with other factors. For example, for every unit increase along the healthcare contact scale there was a 0.11-week (.05–.18-week) or approximately 1-day increase in reported antibiotic use in the last 3 months. Respondents living in rural areas reported an increase of 0.56 weeks (.33­–.79 weeks) or approximately 4 antibiotic use days compared with residents living in urban areas. Compared with adults, children had 0.34 (.17–.50) fewer reported weeks or approximately 2.5 fewer antibiotic use days in the last 3 months (Table 3). Patterns of healthcare contact were also associated with other variables, including the use of an improved toilet facility, which increased the likelihood of healthcare contact compared with use of an unimproved toilet facility (β coefficient, 0.22 [95% CI, .02–.42]). Female participants were predicted to have higher healthcare contact scores than men (β coefficient, 0.51 [95% CI, .32–.70]). Similarly, keeping poultry was associated with other factors. Respondents living in rural areas, compared with urban dwellers, had greater odds of keeping poultry (β coefficient, 5.64 [95% CI, 4.20­–7.58]), as did female participants, compared with male participants (1.40 [1.03–1.90]).

The 500 different random removals indicated that the associations reported in the path model (Table 3) were largely consistent across replications (see Supplementary Table 4 for the range of coefficients across runs and patterns of significance). The overall design effects (changes in variance attributable to the 2-cluster sampling frame) ranged from approximately 1 to 1.4, consistent with limited heteroskedasticity (Supplementary Table 2).

## DISCUSSION

Decreasing the probability of ESCrE colonization in communities relies on identifying behavioral and structural factors (eg, poor sanitation infrastructure) that directly and indirectly contribute to transmission and selection of these organisms. We found that the likelihood of ESCrE colonization increased by about 30% from the lowest to highest levels of healthcare contact; that is, healthcare contact had a direct effect on the probability of ESCrE colonization (see Supplementary Figure 2). We originally surmised that healthcare contact would also increase the duration of use of antibiotics (found to be significant; Figure 1 and Table 3). However, given that antibiotic use was not significantly associated with ESCrE colonization in this analysis, the direct effect of healthcare contact on antibiotic use did not contribute to the likelihood that using antibiotics would increase ESCrE colonization. Both regional-level analysis [22] and other household-focused studies of colonization have failed to find an antibiotic use effect. For example, a study in northern Tanzania found that the strongest correlate for colonization among Maasai households in Tanzania was consumption of raw milk, while other transmission factors were important for Chagga and Arusha households, but not antibiotic use [23].

In an earlier study of Kibera households, no antibiotic use effect was detected but evidence suggested that poor sanitation enhances transmission, leading the authors to speculate that poor sanitation overwhelms effects from individual antibiotic [24]. In Guatemala, a significant interaction effect was detected between household hygiene and antibiotic use whereby poor hygiene likely increased microbial transmission that obscured the effects of individual antibiotic use [25]. While these studies involved different contexts, all relied on self-reported measures of antibiotic use (including the current study) that may have been biased by poor recall or lack of information on antibiotic exposure. If this occurred, there would be a commensurate reduction in the power to detect the effects of antibiotic use on ESCrE colonization. In other contexts, antibiotic use has been a risk factor for colonization with antibiotic resistant bacteria in community-dwellers [26, 27].

The direct effect of healthcare contact on the odds of ESCrE colonization suggests the possibility that transmission of resistant bacteria occurs when respondents visit healthcare facilities either as patients themselves or accompanying a family member for both medical and nonmedical reasons. However, it is also possible that people visiting healthcare locations are more likely to be colonized with ESCrE a priori. For example, people with long-term illness and likely frequent antibiotic exposure (eg, those living with human immunodeficiency virus) may visit healthcare facilities more frequently than their counterparts. Furthermore, if some illnesses are disproportionately associated with ESCrE (eg, diarrhea-causing *Escherichia coli*), the population of healthcare contact might be biased toward individuals who are colonized with ESCrE. Distinguishing between these alternative mechanisms may require a longitudinal study design that is not limited to enrolling presumptively healthy individuals.

We found that keeping poultry at the household was associated with a 57% increase in the odds of ESCrE colonization. Within the city of Nairobi (inclusive of Kibera), a 2022 study [28] examined whole-genome sequences from *E. coli* strains colonizing humans and domestic poultry in 99 households and identified 10 genetically matched strain pairs between people and domestic animals (8 from chickens, turkeys, and a duck). Given that the strains were randomly picked without selective enrichment, finding matches among a genetically diverse species of bacteria is consistent with a relatively high degree of strain sharing between poultry and poultry tenders.

Notably, the Nairobi-based study highlighted above [28] also found evidence that agricultural antibiotic use likely influences the antimicrobial resistance patterns in *E. coli* isolated from chickens. The genetic diversity of antimicrobial resistance genes was lower in poultry isolates than in isolates from other hosts, consistent with purifying selection that enriches populations of selected resistance genes and genes associated by genetic linkage. A survey of veterinary antibiotic suppliers in Nairobi found that while diverse antibiotics are sold to poultry farmers (mostly sulfonamides, tetracyclines, and macrolides), cephalosporins and other β-lactam antibiotics are rarely purchased [29].

Consequently, if veterinary antibiotic use is an indirect factor affecting ESCrE transmission to people through poultry, the mechanism of selection may involve a process of coselection from noncephalosporin antibiotics either at the farm level or at hatchery farms that sell chick stock to local poultry owners. For example, 93% of ESCrE isolates collected from our study participants were also resistant to sulfonamides [16]. If this is representative of ESCrE isolates found in chickens from this community, then using sulfa antibiotics would selectively favor sulfa-resistant strains, of which a large proportion would be ESCrE. These findings, along with previous work [28, 29], support the need to ascertain where selection is occurring and to introduce economically and sex- and locality-contextual practices to help reduce the demand for antibiotics.

Variables predictive of healthcare contact and poultry keeping represent indirect factors affecting the probability of ESCrE colonization. Women were more likely than men to visit hospitals and clinics. This sex bias is consistent with research on healthcare contact dynamics where women are generally more likely to seek medical services [30], although the character of this bias can vary cross-culturally [31] and also with the age and morbidity levels of individuals seeking care [32]. In addition, female participants in our study communities were usually the household's primary caregiver and were consequently more likely to accompany or take dependents to healthcare facilities. The sex bias in healthcare seeking may also have been patterned by antenatal care, although use of these services is comparatively limited in Kenya owing to accessibility, knowledge, and cultural barriers [33, 34].

Access to improved toilet facilities had a direct yet counterintuitive effect on healthcare contact with increased risk of ESCrE colonization. When we investigated this in more detail, the relationship held for both urban and rural settings, but we suspect that the mechanisms differ by setting. In rural settings, improved toilet facilities may be an indicator of household wealth and wealthier households may be able to seek healthcare more frequently. Contrastingly, in Kibera, improved toilet facilities included both flush and slab toilets and are shared between families, which may provide a greater risk of contact transmission [35].

Several variables with potential direct and indirect effects on ESCrE colonization were likely influenced by wealth, but no measures of individual or household wealth (eg, savings, salaries, or assets) were collected. Wealth, for example, is related to access to improved toilet facilities [36, 37] and healthcare contact [38], collectively illustrating potential trade-offs between poverty and increased likelihood of ESCrE colonization within households. Likewise, more research on how gender dynamics influence the selection and transmission of ESCrE is needed because gender-based roles likely affect the introduction and transmission of ESCrE within households. These missing variables, along with household density (people per square meter), may have added important demographic, socioeconomic, and cultural dimensions to inform interventions [39] that target the multiple and interacting factors patterning antimicrobial resistance within communities.

For healthcare contact, a significant relationship with ESCrE colonization was evident after combining hospital and clinic visitation for both medical and nonmedical reasons (4 variables total). By themselves, these factors were not statistically significant, suggesting the presence of threshold effects (ie, a certain degree of healthcare contact is needed before impacts on prevalence can be detected) or an issue of statistical power. In support of the former, a bar plot of healthcare contact ranges on ESCrE does show an observable increase only at the highest levels of contact (see Supplementary Figure 3), although the plot does not control for the impacts of other variables.

Considering these results, more investigation is needed on how healthcare visits (eg, where in the facility patients are attended by health professionals) may affect transmission, especially within healthcare clinics, which have received far less attention than hospitals. While our analysis suggests the potential for causality for hypothesized relationships between sex/gender, wealth, healthcare and ESCrE colonization, a longitudinal study design is needed to identify and confirm causal inferences. In addition, treatment for acute infectious diseases is provided at no cost for participants in our sample, so it is likely that the average number of healthcare contacts and antibiotic use may be higher in this population than Kenya as a whole. Finally, because we restricted our analysis to a single member of a household and did not collect samples from multiple household members, we were unable to determine whether the colonization status of the included individual was significantly associated with the colonization status of other household members [40].

In conclusion, we found that factors that are likely linked to ESCrE transmission (visiting healthcare facilities and keeping poultry) appear to be more important in our study communities than direct selection of resistant organisms from antibiotic use. Using a path analytic approach enabled us to specify how these transmission-related factors may be patterned by underlying characteristics. Transmission-related factors were associated with demographic characteristics, including sex and rural/urban residence, whose impacts are influenced by varying sociocultural, economic, and historical contexts. Consequently, mitigation strategies for antimicrobial resistance in communities may be most effective if focused on the underlying factors that promote transmission of resistance.

## Supplementary Data

Supplementary materials are available at *Clinical Infectious Diseases* online. Consisting of data provided by the authors to benefit the reader, the posted materials are not copyedited and are the sole responsibility of the authors, so questions or comments should be addressed to the corresponding author.

## Supplementary Material

ciad223_Supplementary_Data

## Notes

***Acknowledgments.*** Assistance and support were provided by community members, community leaders, county administration (Nairobi and Siaya counties, Kenya), the Ministry of Health–Kenya, the Diagnostics and Laboratory Systems Program (Kisumu, Kenya), and the University of Nairobi Institute of Tropical and Infectious Diseases Laboratory (Nairobi, Kenya). Jennifer Horton provided additional laboratory support.

***Financial support***. This work was supported by the US Centers for Disease Control and Prevention (grant U01GH002143-05).

***Supplement sponsorship.*** This article appears as part of the supplement “The Evolving Challenges of Antibiotic Resistance in Low- and Middle-Income Countries: Priorities and Solutions,” sponsored by the U.S. Centers for Disease Control and Prevention, and Health Security Partners.
