# Supplementary material for: Risk Factors for Colonization With Multidrug-Resistant Bacteria in Urban and Rural Communities in Kenya: An Antimicrobial Resistance in Communities and Hospitals (ARCH) Study
Source: Clin Infect Dis. 2023 Jul 5;77(Suppl 1):S104–10. doi: 10.1093/cid/ciad223 (PMC10321691; doi:10.1093/cid/ciad223)
Supplement: ciad223_Supplementary_Data [file ciad223_supplementary_data.zip › Community risk factor supplement_ARCH-Kenya-corrected.docx]

Supplement

**Risk factors for carriage of multidrug-resistant bacteria in communities in Kenya: An Antibiotic Resistance in Communities and Hospitals (ARCH) study**

Mark A. Caudell^1^, Charchil Ayodo^2^, Teresa Ita^2^, Rachel M. Smith^3^, Ulzii-Orshikh Luvsansharav^3^, Ashley R. Styczynski^3^, Brooke M. Ramay^1,4^, Samuel Kariuki^5^, Guy H. Palmer^1,2.6^, Douglas R. Call^1^, and Sylvia Omulo^1,2,6^

^1^Paul G. Allen School for Global Health, Washington State University, Pullman, WA, USA, ^2^Washington State University Global Health-Kenya, Nairobi, Kenya, ^3^Division of Healthcare Quality Promotion, U.S. Centers for Disease Control and Prevention, Atlanta, USA, ^4^Center for Health Studies, Universidad del Valle de Guatemala, ^5^Kenya Medical Research Institute, Nairobi, Kenya, ^6^University of Nairobi Institute of Tropical Infectious Diseases, Nairobi, Kenya.

**Table S1.** Hypothesized direct and indirect relationships between variables included in the path model (Fig. 2 and Fig. S1) and the probability of ESCrE colonization. Direct effects occur when a variable is directly predictive of ESCrE colonization. Indirect effects occur when a variable has the potential to influence a direct effect via an intermediary variable.

| **Variable** | **Definitions and rationale** |
| --- | --- |
| **ESCrE** | **Definition** – this is a dependent, binary variable that represents whether an individual is positive or negative for colonization with ESCrE bacteria. 0 = participant stool was negative for extended-spectrum cephalosporin-resistant Enterobacterales (ESCrE), 1 = participant stool was positive for ESCrE. Positive/negative confirmed by Vitek2® assay. |
| **Healthcare contact** | **Definition** – A continuous scaled variable combining answers to four healthcare contact questions^a^, including how many times an individual visited a hospital or clinic for medical or non-medical reasons within the last 6 months. Responses to the four healthcare contact variables were summed to represent how often an individual visited clinics and hospitals in the past 6 months. For the four variables that were summed, responses included 0 = never, 1 = 1-2 times, 2 = 3-4 times, and 3 = ≥5 times, so that 12 was the maximum possible value for the healthcare contact variable (see Table S2).  **Direct** – Healthcare contact could lead to greater chances of contact-dependent and nosocomial transmission to people in health care environments [1–5].  Healthcare contact 🡪 ESCrE  **Indirect** – Patterns of healthcare contact might lead to a greater probability of people taking antibiotics as prescribed or advised by health care professionals, which in turn could lead to a greater probability of colonization with ESCrE [6,7].  Healthcare contact 🡪 Antibiotic use 🡪 ESCrE |
| **Keep poultry** | **Definition** – A binary variable indicating if an individual’s household kept poultry (= 1) or not (= 0).  **Direct** – fecal-oral transmission (which could include ESCrE organisms) can occur through direct contact with animals, and poultry, in particular, have been implicated as a source of antimicrobial-resistant bacteria in Nairobi [8]  Keeping poultry 🡪 ESCrE  **Indirect** – keeping poultry might lead to increased risk of enteric disease via fecal-oral transmission and therefore indirectly impact the probability of ESCrE colonization through healthcare contact practices behaviors or antibiotic use  Keeping poultry 🡪 Healthcare contact 🡪 ESCrE  Keeping poultry 🡪 Antibiotic use 🡪 ESCrE |
| **Antibiotic use** | **Definition** – A continuous variable indicating the number of weeks a respondent reported they used antibiotics in the last 3 months.  **Direct** – antibiotic use could select directly for ESCrE organisms via use of cephalosporin antibiotics, or via co-selection by antibiotics that are hydrolyzed by cephalosporin-resistance enzymes (e.g., CTX-M-15 and ampicillin [9], or through co-selection of resistance traits that are genetically linked to cephalosporin resistance on plasmids or chromosomes.  Antibiotic use 🡪 ESCrE  **Indirect** – we did not postulate any indirect effects of antibiotic use on other variables in the model. |
| **Water source**  **Improved**  **vs.**  **Unimproved** | **Definition** – Improved (= 1) or unimproved (= 0) water sources as defined by the Joint Monitoring Program for Water Supply and Sanitation [10].  **Direct** – if ESCrE bacteria are present in drinking water, then we assume that direct transmission and colonization by ESCrE is more likely with unimproved water sources  Water source 🡪 ESCrE  **Indirect** -- Enteric illness from contaminated water could lead to increased healthcare contact behaviors or to individuals self-medicating with antibiotics  Water source 🡪 Healthcare contact 🡪 ESCrE  Water source 🡪 Antibiotic use 🡪 ESCrE |
| **Toilet**  **Improved**  **vs.**  **Unimproved** | **Definition** – Improved (= 1) or unimproved (= 0) toilet sources. Post-205 JMP definitions were used [10] but the categories of “improved limited” and “improved” were combined given most toilets classified as “improved” were shared.  **Direct** – If ESCrE bacteria are present in human stool, then unimproved toilet facilities might be a direct source of transmission to people.  Toilet 🡪 ESCrE  **Indirect** – Enteric illness, contracted due to poor toilet facilities, could contribute to increased healthcare contact practices or to individuals self-medicating with antibiotics  Toilet 🡪 Healthcare contact 🡪 ESCrE  Toilet 🡪 Antibiotic use 🡪 ESCrE |
| **Rural/urban** | **Definition** – Household was located in urban (= 0) or rural (= 1) setting.  **Direct** – people from urban landscapes tend to harbor a higher prevalence of antimicrobial-resistant bacteria [11,12].  Rural/urban 🡪 ESCrE  **Indirect** – people in rural areas are more likely to raise poultry compared to urban dwellers, and there is a possibility that unmeasured factors related to urban or rural environments could contribute to greater illness and hence more healthcare contact or differential use of antibiotics (multiple potential paths, Fig. S1). |
| **Male/female** | **Definition** – 0 = male, 1 = female.  **Direct** – given that health care decisions in households tends to fall to women before men, there is an unknown likelihood that sex could have a direct impact on the probability of ESCrE colonization  Male/female 🡪 ESCrE.  **Indirect** – women are more likely to seek health care for themselves and to accompany and care for family members [13] presenting an unknown likelihood that sex could have an indirect impact on the probability of ESCrE colonization through health seeking behaviors or antibiotic use. Because poultry-raising can be home-based, it is more likely that women tend poultry than men [14] (multiple potential paths, Fig. S1). |
| **Adult/child** | **Definition** – 0 = adult, 1 = child.  **Direct** – given that children are potentially more likely to develop enteric illness [15–17], there is an unknown likelihood that age could have a direct impact on the probability of ESCrE colonization  Adult/child 🡪 ESCrE  **Indirect** -- given that the diversity of enteric illnesses that affect children [18], there is an unknown likelihood that age could have a direct impact on the probability of ESCrE colonization through healthcare contactor antibiotic use. Keeping of poultry, as with other household/agricultural activities, may be patterned by age (multiple potential paths, Fig. S1). |

**Figure S1. Path model.** Statistically significant (P < 0.05) relationships are indicated by red arrows. For example, the directional arrow between the healthcare contact scale and ESCrE indicates a significant relationship between healthcare contact and ESCrE. Non-significant relationships are indicated by gray arrows.

**Table S2. Variables comprising healthcare contact scale and corresponding ordinal distributions.**

| Healthcare contact^a^ | Not  once | 1-2 times | 3-4 times | 5 or more times |
| --- | --- | --- | --- | --- |
|  | %  (N) | %  (N) | %  (N) | %  (N) |
| Adult/child respondent visited hospital for medical care in last 6 months | 72.99 (1251) | 20.77 (356) | 4.32 (74) | 1.93  (33) |
| Adult/child respondent visited hospital for reasons other than medical care in last 6 months | 69.12 (1184) | 22.53 (386) | 4.5  (77) | 3.85  (66) |
| Adult/child respondent visited clinic for medical care in last 6 months | 73.48 (1258) | 17.46 (299) | 4.96 (85) | 4.09  (70) |
| Adult/child respondent visited clinic for reasons other than medical care in last 6 months | 53.33 (913) | 35.51 (608) | 6.25 (107) | 4.91  (84) |
| Household members visited hospital for medical care in last 6 months | 58.25  (999) | 27.06  (464) | 9.56  (164) | 5.01  (86) |
| Household members visited hospital for reasons other than medical care in last 6 months | 53.47  (917) | 31.02  (532) | 8.69 (149) | 6.59  (113) |
| Household members visited clinic for medical care in last 6 months | 31.55  (541) | 35.51  (609) | 19.59 (336) | 13.06  (224) |
| Household members visited clinic for reasons other than medical care in last 6 months | 57.55  (987) | 26.30  (451) | 8.51  (146) | 7.35  (126) |
| **^a^Healthcare contact questions:**  **Adult or Adult responding for child**  In the past 6 months, did you visit a hospital to receive medical care?  In the past 6 months, did you visit a hospital for reasons other than medical care?  In the past 6 months, did you visit a clinic to receive medical care?  In the past 6 months, did you visit a clinic for reasons other than medical care?  **Reporting on Household members**  In the past 6 months, did HH members visit a hospital to receive medical care?  In the past 6 months, did HH members visit a hospital for reasons other than medical care?  In the past 6 months, did HH members visit a clinic to receive medical care?  In the past 6 months, did HH members visit a clinic for reasons other than medical care? | | | | |

**Table S3. Design effects for the final path model**. Dependent variables in bold. See definitions of DEFF below.

|  | **Linearized coefficient** | **Std. error** | **DEFF** |
| --- | --- | --- | --- |
| **ESCrE** |  |  |  |
| Antibiotic use | -0.047 | 0.035 | 1.286 |
| Healthcare contact | 0.113 | 0.041 | 1.262 |
| Keep poultry | -0.220 | 0.139 | 1.257 |
| Toilet risk | 0.449 | 0.143 | 1.281 |
| Water risk | -0.172 | 0.157 | 1.275 |
| Rural/urban | -0.196 | 0.173 | 1.283 |
| Adult/child | 0.037 | 0.166 | 1.391 |
| Sex | 0.125 | 0.142 | 1.268 |
| constant | -0.749 | 0.295 | 1.238 |
| **Antibiotic use** |  |  |  |
| Healthcare contact | 0.113 | 0.035 | 1.464 |
| Keep poultry | -0.158 | 0.134 | 1.321 |
| Toilet risk | -0.084 | 0.110 | 1.313 |
| Water risk | 0.175 | 0.116 | 1.047 |
| Rural/urban | 0.557 | 0.118 | 1.002 |
| Adult/child | -0.338 | 0.085 | 1.254 |
| Sex | -0.177 | 0.144 | 1.248 |
| constant | -0.060 | 0.205 | 1.190 |
| **Healthcare contact** |  |  |  |
| Keep poultry | 0.512 | 0.097 | 1.216 |
| Toilet risk | -0.188 | 0.102 | 1.234 |
| Water risk | -0.119 | 0.118 | 1.401 |
| Rural/urban | 0.120 | 0.123 | 1.325 |
| Adult/child | -0.415 | 0.097 | 1.299 |
| Sex | 0.222 | 0.102 | 1.215 |
| constant | 5.462 | 0.143 | 1.240 |
| **Keep poultry** |  |  |  |
| Rural/urban | 1.730 | 0.150 | 1.258 |
| Adult/child | -0.288 | 0.193 | 1.364 |
| Sex | 0.333 | 0.157 | 1.215 |
| constant | -0.026 | 0.150 | 1.185 |

DEFF is estimated using [1] as

DEFF = $\frac{\hat{V} \hat{(\theta)}}{\hat{V}_{srswor}\tilde{(\theta)}_{srs}}$

Where $\hat{V} \hat{(\theta)}$ is the design-based estimate of variance for a parameter, $\theta$, and $\hat{V}_{srswor}\tilde{(\theta)}_{srs}$ is an estimate of the variance for an estimator, $\tilde{(\theta)}_{srs}$, that would be obtained from a similar hypothetical survey conducted using SRS without replacement (wor) and with the same number of sample elements, as in the actual survey.

**Figure S2. Impact of individual health seeking on the probability of participant colonization with ESCrE.** The x-axis is the health-seeking scale where the lowest score equals up to four visits to a hospital and/or clinic in the past six months. The highest score on the x-axis represents at least 15 visits. The y-axis is the predicted mean probability of colonization with ESCrE. The shaded area is the 95% confidence interval around the point estimates, which are indicated by dots.

**Table S4. Range of coefficients and number of significant coefficients across 500 runs of the final model with random deletions of households with multiple study participants.** Coefficients for ESCrE and keeping poultry are odds ratios (OR).

|  | **Keep poultry** | | **Healthcare contact** | | **Antibiotic use** | | **ESCrE** | |
| --- | --- | --- | --- | --- | --- | --- | --- | --- |
|  | **OR**  **Range** | **Num**  **sig/500** | **Coef**  **Range** | **Num**  **sig/500** | **Coef**  **Range** | **Num**  **sig/500** | **OR**  **Range** | **Num**  **sig/500** |
| Antibiotic use |  |  |  |  |  |  | 0.892 to 0.995 | 118/500 |
| Healthcare contact |  |  |  |  | 0.052 to 0.126 | 500/500 | 1.041 to 1.165 | 378/500 |
| Keep poultry |  |  | -0.280 to -0.0403 | 94/500 | -0.173 to -0.034 | 0/500 | 1.270 to 1.829 | 484/500 |
| Toilet risk |  |  | 0.147 to 0.379 | 459/500 | -0.256 to 0.0292 | 0/500 | 0.887 to 1.246 | 0/500 |
| Water risk |  |  | -0.239 to -0.060 | 0/500 | 0.0354 to 0.189 | 0/500 | 0.667 to 0.972 | 35/500 |
| Urban/rural | 4.801 to 6.951 | 500/500 | 0.0213 to 0.229 | 0/500 | 0.438 to 0.595 | 500/500 | 0.682 to 0.994 | 9/500 |
| Sex | 1.157 to 1.784 | 338/500 | 0.445 to 0.667 | 500/500 | -0.204 to 0.108 | 0/500 | 0.680 to 1.005 | 61/500 |
| Age | 0.501 to 1.066 | 159/500 | -0.499 to -0.160 | 494/500 | -0.319 to 0.0712 | 355/500 | 0.828 to 1.699 | 19/500 |
| Constant | 0.809 to 1.153 | 0/500 | 5.243 to -5.582 | 500/500 | -0.225 to 0.138 | 0/500 | 0.363 to 0.797 | 195/500 |
| Obs. (n) | 1,415 to 1,418 |  | 1,415 to 1,418 |  | 1,415 to 1,418 |  | 1,415 to 1,418 |  |

**Assessing model fit**

Approaches to determine model fit were constrained by the categorical outcomes, which require estimation of generalized linear response variables and also by the use of clustering effects. Conventional methods of global fit indices, both absolute and relative (e.g., Comparative Fit Index, Root Mean Square Error of Approximation) are based upon maximum likelihood estimation of continuous outcomes [19]. Likelihood ratio tests comparing models as variables/effects are added cannot be used given some exogenous variables become both endogenous and exogenous (healthcare seeking) as we move from null to complete model. Finally, exogenous variables within SEM are often correlated but generalized SEM in Stata is conditional on the observed exogenous variables and cannot be modeled in generalized SEM [20].

**Figure S3. Bar plot of heathcare contact categories by mean prevalence of ESCrE**

**References**

1 Friedmann R, Raveh D, Zartzer E, *et al.* Prospective evaluation of colonization with extended-spectrum beta-lactamase (ESBL)-producing enterobacteriaceae among patients at hospital admission and of subsequent colonization with ESBL-producing enterobacteriaceae among patients during hospitalization. *Infect Control Hosp Epidemiol* 2009;**30**:534–42. doi:10.1086/597505

2 Sikora A, Zahra F. Nosocomial Infections. In: *StatPearls [Internet].* Treasure Island (FL): : StatPearls Publishing 2022.

3 Lax S, Sangwan N, Smith D, *et al.* Bacterial colonization and succession in a newly opened hospital. *Sci Transl Med* 2017;**9**. doi:10.1126/SCITRANSLMED.AAH6500

4 Lax S, Smith D, Sangwan N, *et al.* Colonization and Succession of Hospital-Associated Microbiota HHS Public Access. *Sci Transl Med* 2017;**9**. doi:10.1126/scitranslmed.aah6500

5 McCreesh N, Karat AS, Govender I, *et al.* Estimating the contribution of transmission in primary healthcare clinics to community-wide TB disease incidence, and the impact of infection prevention and control interventions, in KwaZulu-Natal, South Africa. *BMJ Glob Health* 2022;**7**:e007136.

6 Woerther PL, Andremont A, Kantele A. Travel-acquired ESBL-producing Enterobacteriaceae: impact of colonization at individual and community level. *J Travel Med* 2017;**24**:S29. doi:10.1093/JTM/TAW101

7 Harris AD, McGregor JC, Johnson JA, *et al.* Risk Factors for Colonization with Extended-Spectrum β-Lactamase–producing Bacteria and Intensive Care Unit Admission - Volume 13, Number 8—August 2007 - Emerging Infectious Diseases journal - CDC. *Emerg Infect Dis* 2007;**13**:1144–9. doi:10.3201/EID1308.070071

8 Muloi DM, Wee BA, McClean DMH, *et al.* Population genomics of Escherichia coli in livestock-keeping households across a rapidly developing urban landscape. *Nat Microbiol 2022 74* 2022;**7**:581–9. doi:10.1038/s41564-022-01079-y

9 Avillan JJ, Ahmadvand P, Shao-Yeh L, *et al.* Excreted Antibiotics May Be Key to Emergence of Increasingly Efficient Antibiotic Resistance in Food Animal Production. *Appl Environ Microbiol* 2022;**88**. doi:10.1128/AEM.00791-22

10 WHO/UNICEF. JMP Methodology 2017 Update and SDG Baselines, Geneva

11 Subbiah M, Caudell MA, Mair C, *et al.* Antimicrobial resistant enteric bacteria are widely distributed amongst people, animals and the environment in Tanzania. *Nat Commun* 2020;**11**. doi:10.1038/s41467-019-13995-5

12 Walson JL, Marshall B, Pokhrel BM, *et al.* Carriage of Antibiotic-Resistant Fecal Bacteria in Nepal Reflects Proximity to Kathmandu. *J Infect Dis* 2001;**184**:1163–9. doi:10.1086/323647

13 Adedokun ST, Yaya S. Factors influencing mothers’ health care seeking behaviour for their children: evidence from 31 countries in sub-Saharan Africa. *BMC Health Serv Res* 2020;**20**. doi:10.1186/S12913-020-05683-8

14 Guèye EF. Gender issues in family poultry production systems in low-income food-deficit countries. *Am J Altern Agric* 2003;**18**:185–95. doi:10.1079/AJAA200350

15 Lal P, Bansal AK, Aggarwal CS, *et al.* Incidence of diarrhoea and some related environmental and behavioural factors in Jhuggis of Delhi - PubMed. *Indian J Public Health* 1996;**40**:35–7.

16 Kotloff KL, Blackwelder WC, Nasrin D, *et al.* The Global Enteric Multicenter Study (GEMS) of Diarrheal Disease in Infants and Young Children in Developing Countries: Epidemiologic and Clinical Methods of the Case/Control Study. *Clin Infect Dis* 2012;**55**:S232-45. doi:10.1093/cid/cis753

17 Mashoto KO, Malebo HM, Msisiri E, *et al.* Prevalence, one week incidence and knowledge on causes of diarrhea: household survey of under-fives and adults in Mkuranga district, Tanzania. *BMC Public Health* 2014;**14**. doi:10.1186/1471-2458-14-985

18 Liu J, Platts-Mills JA, Juma J, *et al.* Use of quantitative molecular diagnostic methods to identify causes of diarrhoea in children: a reanalysis of the GEMS case-control study. *The Lancet* 2016;**388**:1291–301. doi:10.1016/S0140-6736(16)31529-X

19 Kline RB. *Principles and practice of structural equation modeling*. New York: : The Guilford Press 2010.

20 StataCorp. Stata Lasso Reference Manual: Release 17. College Station, Texas: : StataCorp LLC
