# Supplementary figures and images for: Risk Factors for Colonization With Multidrug-Resistant Bacteria in Urban and Rural Communities in Kenya: An Antimicrobial Resistance in Communities and Hospitals (ARCH) Study
Source: Clin Infect Dis. 2023 Jul 5;77(Suppl 1):S104–10. doi: 10.1093/cid/ciad223 (PMC10321691; doi:10.1093/cid/ciad223)

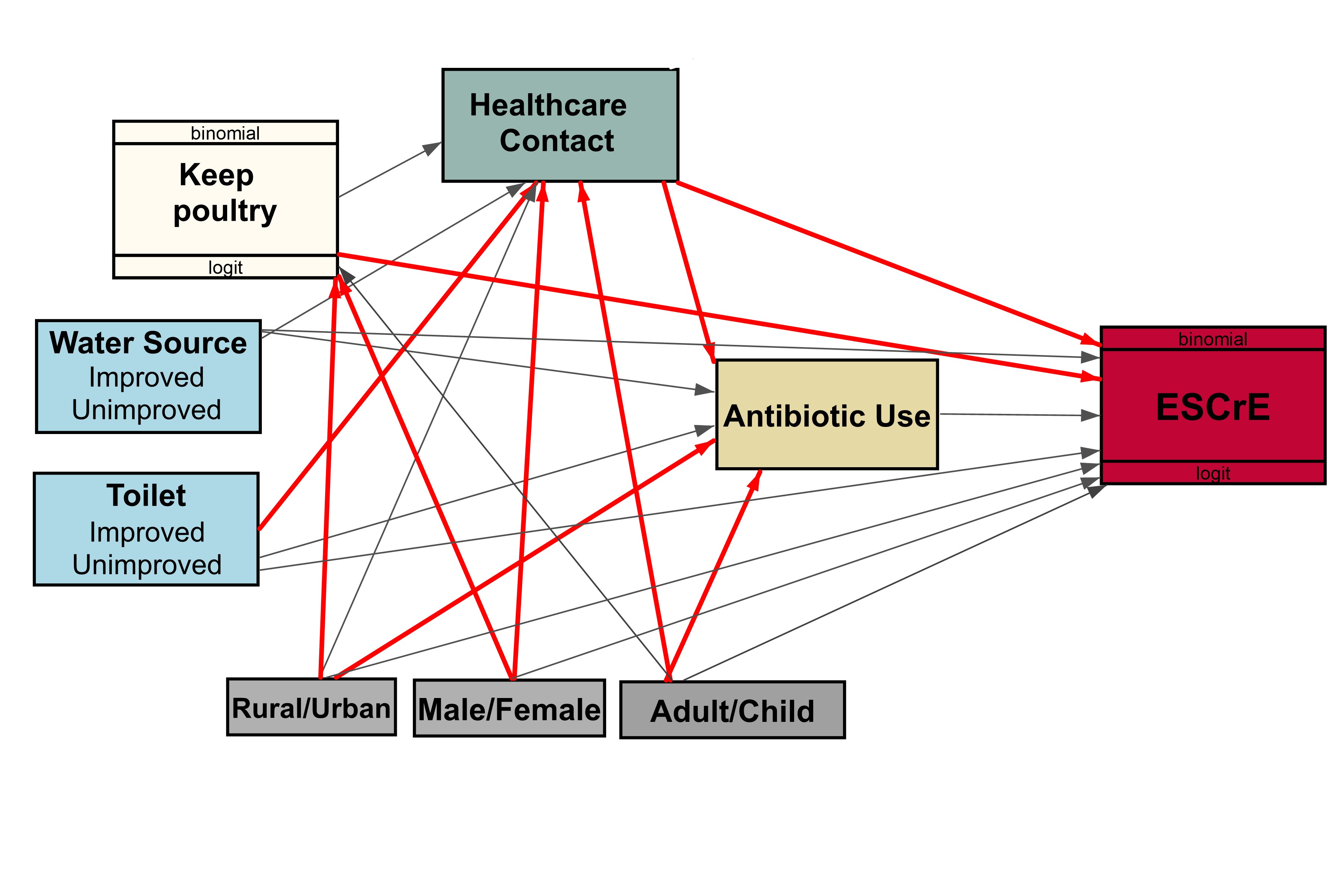

Supplement: ciad223_Supplementary_Data [file ciad223_supplementary_data.zip › Figure S1_600 dpi.jpg]

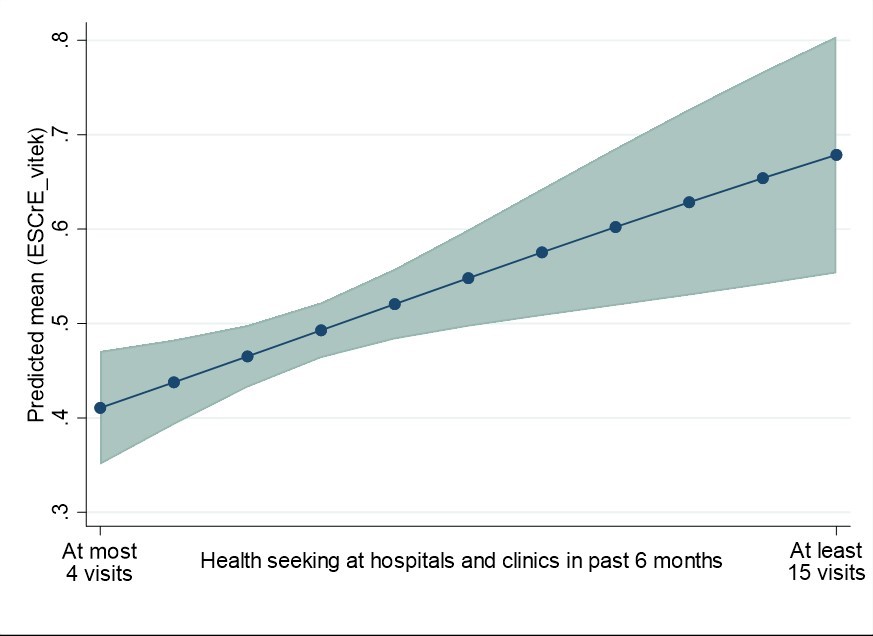

Supplement: ciad223_Supplementary_Data [file ciad223_supplementary_data.zip › Figure S2_600 dpi-corrected.jpg]

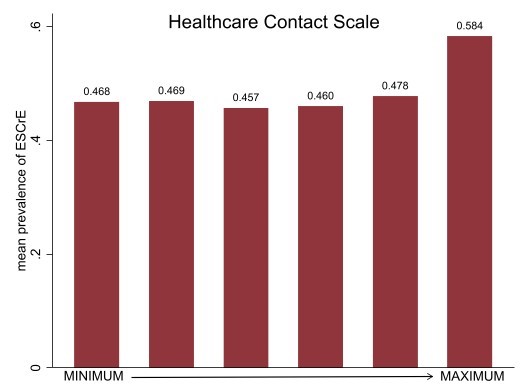

Supplement: ciad223_Supplementary_Data [file ciad223_supplementary_data.zip › Figure S3_600 dpi.jpg]
